# The effective screening tools for detecting hearing loss in elderly population: HHIE-ST Versus TSQ

**DOI:** 10.1186/s12877-020-01996-9

**Published:** 2021-01-09

**Authors:** Nichtima Chayaopas, Pornthep Kasemsiri, Panida Thanawirattananit, Patorn Piromchai, Kwanchanok Yimtae

**Affiliations:** 1 Department of Otorhinolaryngology, Faculty of Medicine, Srinagarind Hospital, Khon Kaen University, Khon Kaen, 40002 Thailand; 2 Khon Kaen Ear, Hearing and Balance Research Group, Faculty of Medicine, Khon Kaen University, Khon Kaen, Thailand

**Keywords:** Hearing loss, Hearing screening, Hearing Handicap Inventory in older adults, Hearing rehabilitation

## Abstract

**Background:**

Globally increasing number of elders is concerned. Hearing loss process in older adults cannot be avoided. An effective screening tool for hearing loss is essential for proper diagnosis and rehabilitation, which can improve QOL in older adults.

**Methods:**

This prospective-diagnostic test study evaluates the diagnostic value of Thai version of the Hearing Handicap Inventory for Elderly Screening (HHIE-ST) and the Thai Single Question (TSQ) surveys in screening hearing disability in 1109 Thai participants aged 60 years and older in communities in four provinces in Thailand. The HHIE-ST consisted of 10 selected questions from the validated HHIE-Thai version. A TSQ survey was developed to have the same meaning as an English Single Question survey. The participants answered both questionnaires, and a standard audiometry test assessed with air conduction from 250 to 8000 Hz was included as a gold standard.

**Results:**

The prevalence of hearing disability was 38.34%. The HHIE-ST achieved a sensitivity of 88.96% (95% CI 85.77–91.64) and specificity of 52.19% (95% CI 48.24–56.13) for diagnosis hearing disability in Thai older adults, whereas the TSQ yielded a sensitivity of 88.73% and a specificity of 55.93%. A combined test including the HHIE-ST and TSQ achieved better performance with sensitivity of 85.29% and specificity of 60.13%.

**Conclusions:**

Either the HHIE-ST or the TSQ is a sensitive and useful tool for screening hearing disability in Thai older adults. Using the HHIE-ST together with the TSQ resulted in a better screening tool for detecting moderate hearing loss older adults who will benefit and recommended for hearing rehabilitation.

**Trial registration:**

The study is registered with the following number in the Thai Clinical Trials Registry: TCTR20151015003. Date of registration October 14, 2015.

## Background

Thailand, as well as most global nations, is now becoming an aging society given the rising number of older adults. In 2014, the Thai national health survey reported that 14.9% of the Thai population was over 60 years old and the prevalence of hearing disability in this population was 24.4% [1]. Most hearing impairment in the older adult is caused by presbycusis, which can be mild or progressive [2, 3]. Therefore, unrecognized older adults do not receive proper diagnosis and rehabilitation. Many studies have shown that hearing disability impacts quality of life, increases occupational accidents, leads to mental and cognitive impairments and increases the mortality rate in the older adult population [4–10].

The standard method for the diagnosis of hearing impairment is standardized pure tone audiometry. The diagnostic criteria for diagnosing hearing impairment are a pure tone average hearing level in the better ear (PTA BE) of greater than 25 dB at 500, 1000, 2000 and 4000 Hz [11]. The Thai National Health Security Office (NHSO) defines hearing disability and provides rights, including free hearing aid, for individuals with bilateral hearing loss with a PTA BE greater than 40 dB on standardized pure tone audiometry.

To achieve the standard diagnosis of and rehabilitation for hearing disability, a large budget, as well as time and expertise, are needed to perform the audiometric test. Due to the limited number of health care providers in many countries, including Thailand, it is difficult to access health services in rural areas, especially those used for hearing screening, diagnosis, and rehabilitation. Although in recent years, there has been the development of mobile or tablet-based hearing screening applications [12], none of them is in Thai. Other challenges are the ability of older people to adopt the new technology, especially in the low-educating community in rural areas, and the availability in the lower-income country. Self-reports are therefore a useful, quick and inexpensive method for screening these types of handicaps in large-scale populations.

Several simple clinical tests have been proposed as methods for screening hearing loss. Whispered voice was one method where examiners whisper words from behind at varying distances, but the results of these tests are highly variable among examiners, possibly due to differences in the loudness of their whispering [13–15]. A tuning fork test, including either the Weber or Rinne test, is another method that has been proposed for screening hearing loss. However, factors that are difficult to control, such as the application of force on the mastoid process and a high false-positive rate, make this test unfavorable for clinical screening [16–20].

Several questionnaires have been developed and used as instruments for assessing hearing disability and handicap; these include the Hearing Handicap Inventory for Elderly (HHIE), the Hearing Handicap Inventory for Elderly Screening Version (HHIE-S), A Single Question (Do you feel you have a hearing loss?) test and Three Single Questions and a 5-Minute Hearing Test [21–26]. These questionnaires have been translated and validated in many languages all over the world [27–31].

The Three Single Questions test has not been tested to determine its diagnostic value for screening hearing impairment in older subjects. The 5-Minute Hearing Test is composed of 15 questions with a wide range of sensitivities and specificities for screening hearing impairment in older subjects. The full version of HHIE has great specificity for identifying hearing impairment in older subjects, but it contains a total of 25 questions and is therefore not suitable as a screening tool due to the time required to complete it.

The Hearing Handicap Inventory for Elderly Screening Version (HHIE-S) has been validated for screening hearing impairment in older subjects, is used as a study tool in various cultures and languages, and results related to its validity and sensitivity are acceptable [27, 29–32]. This questionnaire consists of 10 simple questions, and it is convenient for health care providers or even the general public to complete the test within a few minutes [33, 34]. A full version of HHIE has already been translated and validated in Thai (HHIE-Thai) and shows good intraclass correlation (ICC = 0.63) [35]. As previously mentioned, the HHIE-S was chosen for our study.

The aim of this study was to assess and compare the performance of the Thai version of the HHIE-S instrument (HHIE-ST) and a Thai Single Question (TSQ) test in identifying older subjects with hearing loss against results obtained in measuring hearing loss using PTA, which is the gold standard in Thais over 60 years old.

## Methods

This study is a prospective diagnostic study conducted among older adult Thai subjects in four northeastern provinces, including Khon Kaen, Mahasarakham, Chaiyaphum, and Udon Thani from July 2015 to June 2016. The only inclusion criteria were volunteer Thais over 60 years old. The exclusion criteria were individuals who could not undergo a standard audiometry test, including those with profound otorrhea or difficulty in sitting or responding to sound and persons who could not cooperate with the test due to mental problems.

The HHIE-ST was developed using 10 questions from the validated Thai version (HHIE-Thai) according to the original version of the HHIE-S. For the Thai Single Question (TSQ), we used the question in “The 2012 Disability Survey” [36], which was independently translated from Thai into English by 2 translators, with a final consensus achieved to correspond to the meaning of the question in A Single English Question, which was used as a reference in this study.

The sample size was calculated using 77% for specificity [37] and 80% for sensitivity [25] with a 5% deviation. A total of 1115 subjects were required in this study. The study was conducted in the outpatient department of the otorhinolaryngology clinic of Srinagarind Hospital and the outpatient departments of hospitals in 4 provinces. Informed consent was obtained from all volunteers by a member of the research team before data collection was conducted.

The following demographic data were collected: age, sex, underlying diseases, and educational level. An otologic examination was performed by an otorhinolaryngologist. No intervention was allowed before the hearing assessment was performed. PTA was assessed by an audiologist in a sound-proof booth using a calibrated diagnostic audiometer for hearing thresholds for air conduction from 250 to 8000 Hz.

The audiometry results were interpreted according to WHO classification (1991) using the PTA BE at 500, 1000, 2000 and 4000 Hz. A PTA BE ≤ 25 dB indicated “normal hearing”, a PTA BE 26–40 dB indicated “mild impairment”, a PTA BE 41–60 dB indicated “moderate impairment”, a PTA BE 61–80 dB indicated “severe impairment”, and a PTA BE > 81 dB indicated “profound impairment, including deafness” [11].  High-frequency hearing loss was defined as a PTA at 3000, 4000 and 6000 Hz of greater than 25 dB while the PTA of other frequencies was not greater than 25 dB.

The HHIE-ST consists of 10 questions with answers scored as “yes: 4 points”, “sometimes: 2 points”, and “no: 0 points”. The total score was calculated with a cut-off point score of “more than 8” considered to indicate hearing handicap according to the American Speech-Language-Hearing Association (ASHA) draft guidelines [38]. The TSQ should be answered with “yes” or “no”. Both questionnaires were performed separately.

For volunteers who could not read, the interview was conducted by researchers. All data collection processes were performed on the same day, and the operators were blinded to the results of each process.

We used the Chi-square test to analyze categorical data. The sensitivity, specificity, positive likelihood ratio, and negative likelihood ratio were calculated using STATA software version 10 (Stata Corp, Texas, USA).

This study was approved by the Office of the Khon Kaen University Ethics Committee in Human Research (project number HE581300). The study is registered under the following number at the Thai Clinical Trials Registry: TCTR20151015003.

## Results

**Fig. 1:**
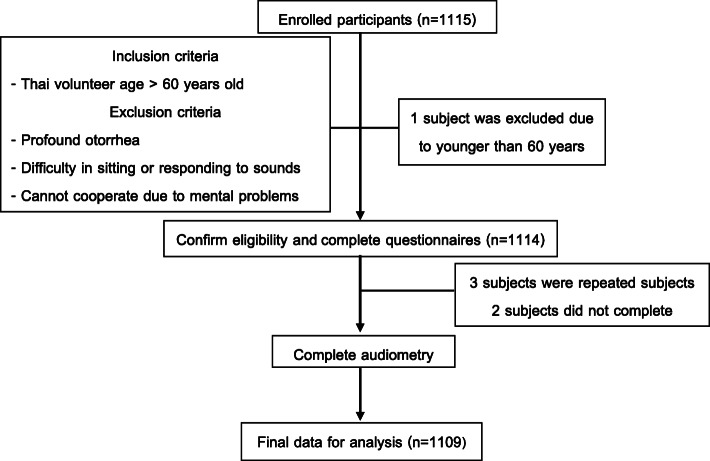
Flow of participant. From 1115 participants that were recruited, 6 subjects were excluded for the following reasons: one was under 60 years old, three were repeated subjects, and two did not complete the audiometry test. Final data for analysis was from 1109 participants

As shown in Fig. 1, 1115 subjects were recruited. We excluded 6 subjects for the following reasons: three subjects were repeated, one was under 60 years old, and two did not complete the audiometry test. Therefore, data on 1109 subjects were analyzed. There were 464 males (41.84%) and 645 females (58.16%). Their ages ranged from 60 to 91 years. Most participants were 60–70 years old (52.84%). In all, 499 subjects were living in urban areas, and 610 were living in rural areas. Educational background was collected, and the majority of subjects had finished primary school (65.64%), while some had a lower than primary school background (3.88%). Hypertension was the most frequent underlying disease and was found in 391 subjects (35.25%), while 290 subjects (18.84%) had diabetes and 10.82% had dyslipidemia. Ear examinations showed that 89.94% were normal ears. Demographic data and more details obtained in the ear examinations are shown in Table 1.

**Table 1:** Participants’ characteristics and demographic data

Characteristic	Demographic data (*N*=1109)	N	%
Sex	Male	464	41.84
	Female	645	58.16
Area	Urban	499	45.00
	Rural	610	55.00
Age, y	Mean (S.D.)	70.75 (7.25)	
	60 - 70	586	52.84
	71 - 80	407	36.70
	> 80	116	10.46
Educational Attained	Lower than primary school	43	3.88
	Primary school	728	65.64
	High school	117	10.55
	Vocational school	44	3.97
	University or higher	177	15.96
Ear Examination	Total	2218	
	Normal	1995	89.94
	Impacted cerumen	100	4.51
	TM perforate/COM	70	3.15
	Retracted TM/OME	32	1.44
	Sclerotic TM	18	0.81
	EAC stenosis	3	0.14
Underlying disease	HT	391	35.25
	DM	209	18.84
	DLP	120	10.82
	Cardiovascular disease	36	3.25
	Autoimmune disease	23	2.07
	CKD	14	1.26
	Vertigo	11	0.99
	Cancer	9	0.81
	Others	180	16.23

**Fig. 2:**
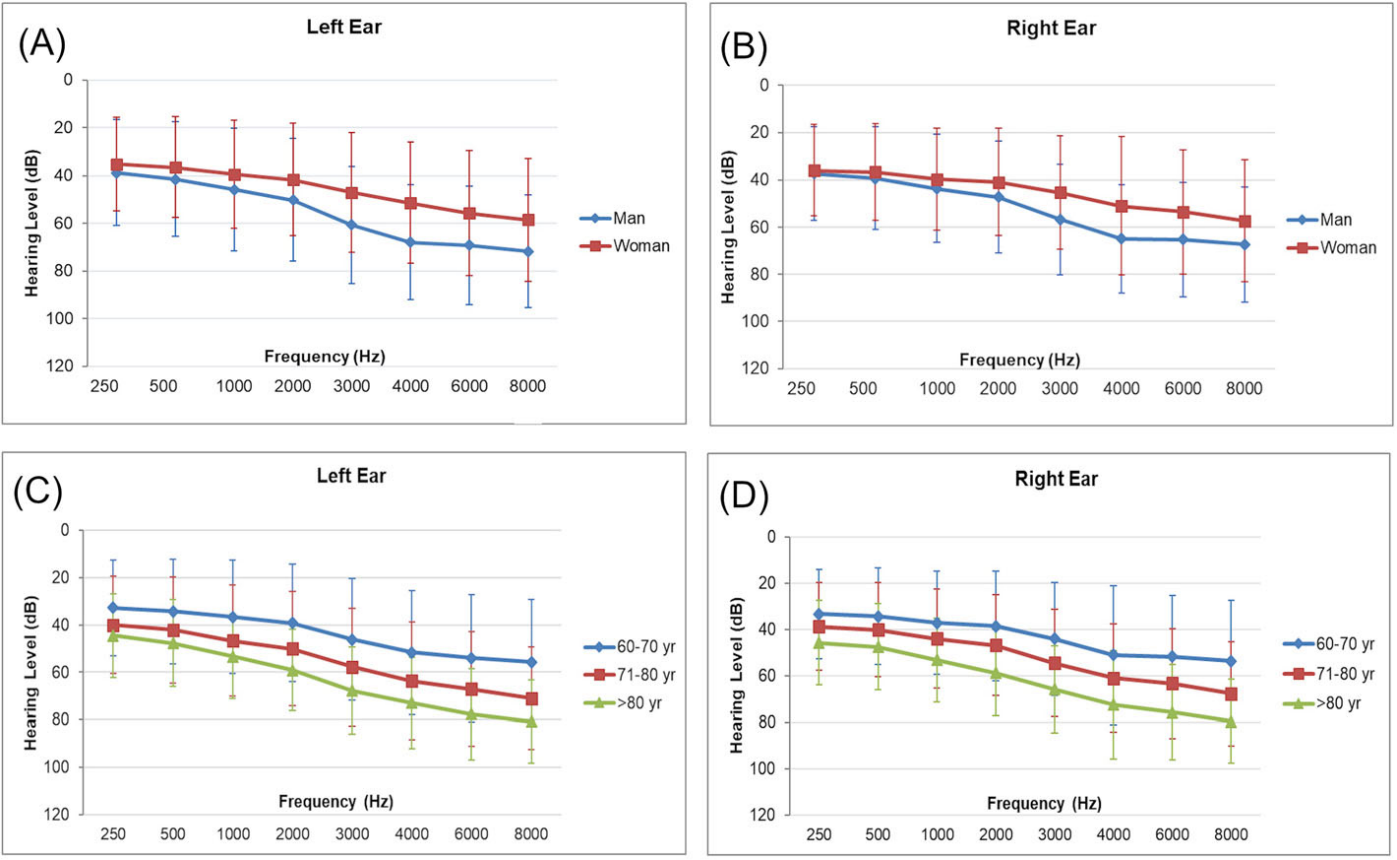
Graphs showing comparisons of hearing levels between the sexes (**a** and **b**) and across age groups (**c** and **d**). Graph (**a**) and (**b**) show hearing characteristic comparing between man (diamond) and woman (square) in left ear (**a**) and right ear (**b**). Graph (**c**) and (**d**) show hearing characteristic comparing between different age groups: 60–70 years old (diamond), 71–80 years old (square) and older than 80 years old (triangle) in left ear (**c**) and right ear (**d**)

Regarding the audiometric results, 276 subjects (24.93%) had a normal PTA (PTA < 25 dB according to WHO criteria) in at least one ear, 166 (15.0%) had high-frequency hearing loss, and 426 (38.34%) had hearing disability. There was a significant difference in hearing level at each frequency between males and females and among the age groups (*p* < 0.001) as shown in Fig. 2. Regarding the PTA of the better-hearing ear at speech frequencies, the prevalence of hearing disability in this study was 38.34%. As for the HHIE-ST, participants with primary school or lower educational status had significantly higher total scores than were found in those who finished high school or higher (*P* < 0.001).

The internal consistency of the HHIE-ST was assessed using Cronbach’s alpha test. The alpha value was 0.94. Pearson’s Moment Correlation Coefficient was 0.8, indicating good correlation.

The sensitivity and specificity of the HHIE-ST (using a cut-off point of 10) for moderate hearing impairment were 88.96% and 52.19%, respectively. The sensitivity and specificity of the TSQ and HHIE-ST for all severities of hearing impairment according to the WHO classification are shown in Table 2.

**Table 2:** The sensitivity and specificity of the TSQ and HHIE-ST (cut-off score at 10) for detecting hearing impairment

	WHO Hearing Level		
	> 25 dB	> 40 dB	> 60 dB
**PTA, n (%)**	834 (75.07)	426 (38.34)	90 (8.10)
**Thai Single Question, n (%)**	584 (52.66)	378 (34.08)	86 (7.75)
**HHIE-ST, n (%)**	608 (54.19)	316 (28.44)	118 (10.64)
**Thai Single Question (TSQ)**			
**Sensitivity (95%CI)**	70.11 (66.80-73.20)	88.73 (85.34-91.57)	95.56 (89.01-98.78)
**Specificity (95%CI)**	65.58 (59.65-71.17)	55.93 (52.11-59.69)	41.81 (38.76-44.90)
**PPV (95%CI)**	86.01 (83.17-88.53)	55.67 (51.84-59.45)	12.67 (10.26-15.40)
**NPV (95%CI)**	42.09 (37.38-46.92)	88.84 (85.47-91.65)	99.07 (97.64-99.75)
**HHIE-ST**			
**Sensitivity (95%CI)**	74.02 (70.85-77.00)	88.96 (85.77-91.64)	100 (96.92-100)
**Specificity (95%CI)**	58.59 (52.75-64.25)	52.19 (48.24-56.13)	38.85 (35.8-41.96)
**PPV (95%CI)**	83.01 (80.07-85.68)	57.87 (54.18-61.50)	16.30 (13.68-19.19)
**NPV (95%CI)**	45.19 (40.15-50.32)	86.49 (82.67-89.75)	100 (99.05-100)
**HHIE-ST or TSQ**			
**Sensitivity (95%CI)**	82.43 (79.62-84.99)	94.27 (91.77-96.19)	100 (96.92-100.00)
**Specificity (95%CI)**	41.75 (36.08-47.59)	37.70 (33.91-41.60)	26.95 (24.20-29.84)
**HHIE-ST and TSQ**			
**Sensitivity (95%CI)**	69.35 (66.04-72.52)	85.29 (81.75-88.37)	96.61 (91.55-99.07)
**Specificity (95%CI)**	68.47 (62.84-73.74)	60.13 (56.19-63.97)	45.27 (42.13-48.44)

To compare the sensitivity and specificity of the HHIE-ST at different cut-off points ranging from 10 to 16, ROC curves were generated, and the AUC was calculated and is shown in Table 3.

**Table 3:** Screening performance of HHIE-ST at different cut-off points

AUC at different severity of hearing loss (95% CI)	HHIE-ST cut-off	True-positive rate (Sensitivity)	Specificity	LR+	LR-
0.74 (0.70–0.77) > 25 dB	( > = 10 )	74.01	58.59	1.79	0.44
	( > = 12 )	70.32	62.96	1.90	0.47
	( > = 14 )	66.50	68.35	2.10	0.49
	( > = 16 )	64.16	73.40	2.41	0.49
0.80 (0.78–0.83) > 40 dB	( > = 10 )	88.96	52.19	1.86	0.21
	( > = 12 )	86.84	57.37	2.04	0.23
	( > = 14 )	83.01	61.91	2.18	0.27
	( > = 16 )	81.95	66.46	2.44	0.27
0.82 (0.80–0.86) > 60 dB	( > = 10 )	100	38.85	1.63	0
	( > = 12 )	100	43.19	1.76	0
	( > = 14 )	98.31	47.73	1.88	0.03
	( > = 16 )	97.46	51.06	1.99	0.04

## Discussion

Hearing loss in older adults, similar to most parts of the world, is not uncommon in Thailand. The prevalence of hearing disability, defined as a PTA > 40 dB in the better hearing ear in elder subjects, has been reported in different parts of the world to be approximately one-third of the older population [2, 39–42]. In Thailand, the prevalence of hearing disability among older subjects was 24.40% in a previous study by Bunnag et al. published in 2002 [1]. However, in our study, we found that the prevalence of hearing disability was higher, at 38.34% (risk difference = 13.90%). The external ear and middle ear pathology could contribute to the increasing of hearing disability prevalence. There were approximately 10 percent of participants in this study who had the external or middle ear pathology in either ear, half of them had PTA > 40 dB in the better hearing ear. Therefore, the otoscopy examination should be encouraged to confirm the diagnosis. The increase in the prevalence of hearing disability in this study could be explained by the differences in study settings and population. Our study was conducted in rural communities where the risk for loud noise exposure from agriculture machines is higher than in urban communities. Relevance to the study by Wang et al. [43] which included the retired staff of an automobile manufactory, who may be more likely to have been exposed to loud noises in their prior environment, we found we have lower prevalence with a higher number of participants.

Presbycusis is the most common cause of hearing loss in the older population [39]. This process slowly progresses; hence, some older adults with hearing loss do not realize the problem and therefore do not seek prompt medical attention. Diagnosis and rehabilitation can be delayed, especially in community-based patients. Earlier studies showed that only 7.5–10% of older adults with hearing disability used hearing aids [42, 44]. Hands reported a reduction in hearing handicap and an increase in overall hearing aid usage in older subjects in their cohort study of routine hearing screening performed with the HHIE-S in the older population [45]. Raising awareness of hearing problems by implementing feasible screening methods should lead to a higher number of older adults receiving proper hearing rehabilitation. This is especially important in people with severe hearing loss (i.e., a PTA ≥ 60 dB) who are more likely to adopt the use of a hearing aid and have reported a higher QOL due to hearing rehabilitation [46–48].

Ensuring that validated questionnaires are adopted and applied across different cultural contexts while retaining the reliability and validity of the original version is a challenge. For our study, we found that the reliability was very good for our Thai version and that our internal consistency was higher than that achieved by the Swedish version and comparable to that of the original version. The Cronbach’s alpha score for the HHIE-ST, the original version, the Japanese version and the Swedish version are 0.94, 0.82, 0.91 and 0.77, respectively [23, 27, 49].

Regarding HHIE-ST scores and demographic data correlations, we found that mean scores were significantly correlated with age group, with older individuals having higher scores (*p* < 0.001). There was no significant difference in HHIE-ST scores between sex or area of residence. However, there were significant differences in the total scores among groups with different levels of education, with higher scores received by participants with lower educational levels (*p* < 0.001). We suggest that this could be related to the ability to understand the true meaning of the questions and the possibility that there are differences in self-awareness between these groups. Average hearing levels were worse in men than in women at every frequency and worsened with age, as expected, although these trends were not statistically significant.

Concerning the diagnostic value of this study, at a cut-off score 10 and a PTA BE higher than 40 dB, the sensitivity of HHIE-ST was 88.96%, slightly higher than that reported by Sindhusake et al [32] (80%) and Tomioka et al [27] (81.30%). The report of specificity was varied from 59 to 92% in other studies in the literature [23, 27, 29–33, 50]. In our study, the specificity was 52.19%. This result may reflect the influence of differences in culture, language, and religion.

Furthermore, a similar study in Thai population was done recently. Judee et al. reported the sensitivity of HHIE-ST at a cut-off score 10 for detecting hearing disability was 69.7% and specificity was 74.9 [51], which is lower sensitivity with higher specificity than in our study. This could be explained by the smaller number of participants than our study, 220 to 1109 participants. And the PTA in Judee et al. study was calculated over the frequencies of 500, 1,000, and 2,000 Hz for the better ear hearing level, while we included 4,000 Hz in the PTA calculation for grading of hearing impairment according to WHO classification in our study.

Given all the reasons outlined above and according to the ROC curves shown in Table 3, we found that the HHIE-ST with the cut-off point 10 is sensitive for detecting Thai older adults with moderate hearing loss who are targeted for hearing rehabilitation.

The diagnostic value of the A Single Question survey has been reported to range from 48–90% for sensitivity and 50–91% for specificity [22, 23, 26, 27, 30, 32, 44, 52–54]. For the TSQ, we obtained a sensitivity of 88.73% and a specificity of 55.93% for detecting moderate hearing loss and a sensitivity of 95.56% and a specificity of 41.81% for screening for severe hearing loss. This result is similar to that found in a Japanese study [27] and the Blue Mountain study [32], which screened for moderate hearing loss; both of these studies also included more than one thousand participants, similar to our study.

We recommend that both the TSQ and the HHIE-ST are sensitive and useful for screening eligible hearing-disabled persons. However, we found that using the HHIE-ST in combination with the TSQ can increase specificity to 60.13% while sensitivity is still as high at 85.29%, as shown in Table 2. This is an effective screening method that could increase the detection rate and raise awareness for hearing rehabilitation in the hearing-disabled population.

The strength of this study is a large number of participants with varying educational levels and urban-rural community status. In addition, we conducted this study in a setting that accurately represents how the screening process would likely be done in a limited resource context. We performed the audiometry test in a sound-proof booth in the quietest area possible instead of using a sound-proof testing room, this could result in some error in the audiometry results. However, we conducted the test according to the WHO hearing measurement guidelines for nonclinical settings [55]. Hence, we believe that our study results should accurately represent the results that would be obtained in a general Thai older population when using these hearing screening tools and should present the least bias. In terms of how better results could be obtained with sensitivity and specificity, we recommend that language modification of a simplified questionnaire may be more appropriate for the Thai lifestyle.

## Conclusions

Both the HHIE-ST and the TSQ are sensitive tools for screening eligible hearing disabled persons. However, a combination including the HHIE-ST with a cut-off score of 10 and the TSQ is a better screening tool for detecting Thai older adults with moderate hearing loss who will benefit and recommended for hearing rehabilitation.

## Data Availability

The datasets used and/or analyzed during the current study are available from the corresponding author on reasonable request.

## Abbreviations

HHIE-ST: Thai version of the Hearing Handicap Inventory for Elderly Screening
TSQ: Thai Single Question
QOL: Quality of life
HHIE-S: Hearing Handicap Inventory for the Elderly – Screening version
PTA BE: Pure tone average hearing level in the better ear
PTA: Pure tone average
ROC: Receiver operating characteristic curve
AUC: The area under the curve

## References

[1] Bunnag C, Prasansuk S, Nakorn AN, Jareoncharsri P, Atipas S, Angsuwarangsee T, et al. Ear diseases and hearing in the Thai elderly population. Part I. A comparative study of the accuracy of diagnosis and treatment by general practitioners vs ENT specialists. J Med Assoc Thai. 2002 May;85(5):521–31. 12188380

[2] Cruickshanks KJ, Wiley TL, Tweed TS, Klein BEK, Klein R, Mares-Perlman JA (1998). Prevalence of hearing loss in older adults in Beaver dam, Wisconsin. The epidemiology of hearing loss study. Am J Epidemiol.

[3] Mulrow CD. Screening for hearing impairment in the elderly. Hosp Pract (Off Ed). 1991;26(2A):79, 83–6. 1899676

[4] Saito H, Nishiwaki Y, Michikawa T, Kikuchi Y, Mizutari K, Takebayashi T (2010). Hearing handicap predicts the development of depressive symptoms after 3 years in older community-dwelling Japanese. J Am Geriatr Soc.

[5] Gopinath B, Hickson L, Schneider J, Mcmahon CM, Burlutsky G, Leeder SR (2012). Hearing-impaired adults are at increased risk of experiencing emotional distress and social engagement restrictions five years later. Age Ageing.

[6] Lodeiro-Fernandez L, Lorenzo-Lopez L, Maseda A, Nunez-Naveira L, Rodriguez-Villamil JL, Millan-Calenti JC (2015). The impact of hearing loss on language performance in older adults with different stages of cognitive function. Clin Interv Aging.

[7] Nondahl DM, Cruickshanks KJ, Dalton DS, Klein BEK, Klein R, Schubert CR, et al. The Impact of Tinnitus on Quality of Life in Older Adults. J Am Acad Audiol. 2007;18(3):257–66. 10.3766/jaaa.18.3.7 17479618

[8] Appollonio I, Carabellese C, Frattola L, Trabucchi M (1996). Effects of sensory aids on the quality of life and mortality of elderly people: a multivariate analysis. Age Ageing.

[9] Clark K, Sowers MR, Wallace RB, Jannausch ML, Lemke J, Anderson CV (1995). Age-related hearing loss and bone mass in a population of rural women aged 60 to 85 years. Ann Epidemiol.

[10] Zwerling C, Whitten PS, Davis CS, Sprince NL (1997). Occupational injuries among workers with disabilities: the National Health Interview Survey, 1985–1994. JAMA.

[11] World Health Organisation. Report of the Informal Working Group on Prevention of Deafness and Hearing Impairment Programme Planning. 1991. p. 1–24.

[12] Livshitz L, Ghanayim R, Kraus C, Farah R, Even-Tov E, Avraham Y (2017). Application-Based Hearing Screening in the Elderly Population. Ann Otol Rhinol Laryngol.

[13] Eekhof JAH, De Bock GH, De Laat JAPM, Dap R, Schaapveld K, Springer MP (1996). The whispered voice: The best test for screening for hearing impairment in general practice?. Br J Gen Pract.

[14] Swan IR, Browning GG (1985). The whispered voice as a screening test for hearing impairment. J R Coll Gen Pract.

[15] Browning GG, Swan IR, Chew KK (1989). Clinical role of informal tests of hearing. J Laryngol Otol.

[16] Burkey JM, Lippy WH, Schuring AG, Rizer FM (1998). Clinical utility of the 512-Hz Rinne tuning fork test. Am J Otol.

[17] Chole RA, Cook GB (1988). The Rinne test for conductive deafness. A critical reappraisal. Arch Otolaryngol Head Neck Surg.

[18] Johnston DF (1992). A new modification of the Rinne test. Clin Otolaryngol..

[19] Stankiewicz JA, Mowry HJ (1979). Clinical accuracy of tuning fork tests. Laryngoscope.

[20] Bagai A, Thavendiranathan P, Detsky AS (2006). Does this patient have hearing impairment?. JAMA..

[21] Ventry IM, Weinstein BE (1982). The hearing handicap inventory for the elderly: a new tool. Ear Hear.

[22] Ferrite S, Santana VS, Marshall SW (2011). Validity of self-reported hearing loss in adults: Performance of three single questions. Rev Saude Publica.

[23] Gates GA, Murphy M, Rees TS, Fraher A (2003). Screening for handicapping hearing loss in the elderly. J Fam Pract.

[24] Koike KJ, Hurst MK, Wetmore SJ (1994). Correlation between the American Academy of Otolaryngology-Head and Neck Surgery Five-Minute Hearing Test and standard audiologic data. Otolaryngol - Head Neck Surg.

[25] Weinstein BE, Ventry IM (1983). Audiometric Correlates of the Hearing Handicap Inventory for the Elderly. J Speech Hear Disord.

[26] Wu HY, Chin JJ, Tong HMH (2004). Screening for hearing impairment in a cohort of elderly patients attending a hospital geriatric medicine service. Singapore Med J.

[27] Tomioka K, Ikeda H, Hanaie K, Morikawa M, Iwamoto J, Okamoto N (2013). The Hearing Handicap Inventory for Elderly-Screening (HHIE-S) versus a single question: Reliability, validity, and relations with quality of life measures in the elderly community, Japan. Qual Life Res.

[28] Yimtae K, Kasemsiri P, Thanawirattananit P, Siripaopradith P (2014). Validation of the thai five-minute hearing test to screen hearing loss in the community. Audiol Neurotol.

[29] Diao M, Sun J, Jiang T, Tian F, Jia Z, Liu Y (2014). Comparison between self-reported hearing and measured hearing thresholds of the elderly in China. Ear Hear.

[30] Deepthi R, Kasthuri A (2012). Validation of the use of self-reported hearing loss and the Hearing Handicap Inventory for elderly among rural Indian elderly population. Arch Gerontol Geriatr.

[31] Salonen J, Johansson R, Karjalainen S, Vahlberg T, Isoaho R (2011). Relationship between self-reported hearing and measured hearing impairment in an elderly population in Finland. Int J Audiol.

[32] Sindhusake D, Mitchell P, Smith W, Golding M, Newall P, Hartley D (2001). Validation of self-reported hearing loss. The Blue Mountains Hearing Study. Int J Epidemiol.

[33] McBride W, Mulrow C, Aguilar C, Tuley M (1994). Methods for Screening Hearing Loss in Older Adults. Am J Med Sci.

[34] Ventry IM, Weinstein BE (1983). Identification of elderly people with hearing problems. ASHA.

[35] Laohasiriwong S, Kasemsiri P, Thanavirathananich P, Yimtae K (2018). Validity and Test-Retest Reliability of Hearing Handicap Inventory for the Elderly Thai Version (HHIE-Thai). Ann Otolaryngol Rhinol.

[36] National statistical office/Ministry of information and communication office. The 2012 DISABILITY SURVEY. 2014.

[37] Lichtenstein MJ, Bess FH, Logan SA (1988). Validation of screening tools for identifying hearing-impaired elderly in primary care [published erratum appears in JAMA 1990 38]. JAMA.

[38] Lichtenstein MJ, Bess FH, Logan SA (1988). Diagnostic Performance of the Hearing Handicap Inventory for the Elderly (Screening Version) Against Differing Definitions of Hearing Loss. Ear Hear.

[39] Gates GA, Mills JH (2005). Presbycusis Lancet.

[40] Jennings CR, Jones NS, Presbyacusis (2001). J Laryngol Otol.

[41] Kim HN, Kim SG, Lee HK, Ohrr H, Moon SK, Chi J (2000). Incidence of presbycusis of Korean populations in Seoul, Kyunggi and Kangwon provinces. J Korean Med Sci.

[42] Lee Jek Chong G, Danker AN, Wong YH, Lim MY (2017). Hearing loss amongst the elderly in a Southeast Asian population - A community based study. Ann Acad Med Singapore.

[43] Wang Y, Mo L, Li Y, Zheng Z, Qi Y (2017). Analysing use of the Chinese HHIE-S for hearing screening of elderly in a northeastern industrial area of China. Int J Audiol.

[44] Gates GA, Cooper JC, Kannel WB, Miller NJ (1990). Hearing in the elderly: the Framingham cohort, 1983–1985. Part I. Basic audiometric test results. Ear Hear.

[45] Hands S (2000). Hearing loss in over-65s: is routine questionnaire screening worthwhile?. J Laryngol Otol.

[46] Manrique-Huarte R, Calavia D, Irujo AH, Girón L, Manrique-Rodríguez M (2016). Treatment for Hearing Loss among the Elderly: Auditory Outcomes and Impact on Quality of Life. Audiol Neurotol.

[47] Aazh H, Prasher D, Nanchahal K, Moore BCJ (2015). Hearing-aid use and its determinants in the UK National Health Service: A cross-sectional study at the Royal Surrey County Hospital. Int J Audiol.

[48] Gallagher NE, Woodside JV (2018). Factors Affecting Hearing Aid Adoption and Use: A Qualitative Study. J Am Acad Audiol.

[49] Öberg M (2016). Validation of the Swedish Hearing Handicap Inventory for the Elderly (Screening Version) and Evaluation of Its Effect in Hearing Aid Rehabilitation. Trends Hear.

[50] Servidoni AB, De Oliveira Conterno L (2018). Hearing loss in the elderly: Is the hearing handicap inventory for the elderly - Screening version effective in diagnosis when compared to the audiometric test?. Int Arch Otorhinolaryngol.

[51] Judee N, Charusripan P (2020). Sensitivity and Specificity of the Hearing Handicap Inventory for Elderly-Screening Thai Version. J Med Assoc Thail.

[52] Voeks SK, Gallagher CM, Langer EH, Drinka PJ (1993). Self-reported hearing difficulty and audiometric thresholds in nursing home residents. J Fam Pract.

[53] Nondahl DM, Cruickshanks KJ, Wiley TL, Tweed TS, Klein R, Klein BE (1998). Accuracy of self-reported hearing loss. Audiology.

[54] Mukari SZMS, Wan Hashim WF (2018). Self-Perceived Hearing Loss, Hearing-Help Seeking and Hearing Aid Adoption Among Older Adults in Malaysia. Ann Otol Rhinol Laryngol.

[55] Franks JR, Goelzer B, Hansen CH, Sehrndt G (2001). Hearing Measurement. Occupational exposure to noise: evaluation, prevention and control.

